# Preview of “Data and its (dis)contents: A survey of dataset development and use in machine learning research”

**DOI:** 10.1016/j.patter.2021.100388

**Published:** 2021-11-12

**Authors:** Nicholas Vincent, Brent Hecht

**Affiliations:** 1 Northwestern University, Evanston, IL, USA

## Abstract

In this preview, we highlight what we believe to be the major contributions of the review and discuss opportunities to build on the work, including by closely examining the incentive structures that contribute to our dataset culture and by further engaging with other disciplines.

## Main text

As technologies incorporating machine learning have become more powerful, profitable, and prominent in society, evidence has been mounting that such systems can amplify existing inequalities and create new harms. For instance, audits of widely used systems ranging from search engines to applications of computer vision have revealed a variety of “problematic behaviors,”^1^ and evidence from practitioners suggests that the undervaluation of data work causes cascading negative effects, such as poor performance.^2^

The machine learning research community has begun to address data-related concerns, for instance through the introduction of “negative impact statements”^3^ and a “dataset track”^4^ at NeurIPS. Various studies have highlighted the importance of documenting data^5^^,^^6^ and issues with models trained on large, previously undocumented datasets.^7^ However, there is still much uncertainty about the path forward; many of the serious concerns raised over the years remain unaddressed in practice.

In this issue of *Patterns*, Paullada and colleagues examine concerns that past and ongoing dataset practices have created “faulty foundations” for machine learning and outline avenues for the machine learning research community to begin to improve the norms of building, designing, inspecting, maintaining, and choosing to use datasets.^8^ The paper—a structured review of dataset practices in machine learning research—argues that machine learning researchers (and researchers in other fields who use machine learning) urgently need to reframe the way they think about machine learning datasets. The paper provides both long-term suggestions for steering the culture of machine learning in a better direction and immediate suggestions on which researchers can act (e.g., data documentation practices).

Focusing on computer vision and natural language processing, Paullada et al. identify three areas of particular concern: the design (i.e., content and structure) of datasets, techniques to explore and improve datasets, and dataset culture. For each of these areas, the review extracts key findings and arguments from relevant studies and makes suggestions for research and practice going forward.

Reviewing issues with the content and structure of popular datasets, the authors highlight concerns regarding representational harms, spurious correlations, and datasets that by their existence legitimize certain problem formulations. This section also summarizes evidence that dataset collection, annotation, and documentation practices leave much to be desired.

Next, the authors review techniques to inspect and algorithmically explore data, emphasizing that such techniques are only helpful when a given dataset is aligned with a well-defined and well-motivated task. This section highlights techniques that have shown to be useful but also reifies that inspection techniques cannot solve fundamental issues with a dataset’s construction or task framing.

Finally, the paper reviews dataset culture; i.e., what are the common practices around data use, storage, and re-use, and what are the implications of these practices? The authors also emphasize the labor conditions of dataset production and the role of legal institutions in changing dataset culture.

Taken together, these contributions will be of interest to the many scholars invested in the growing debate about improving dataset practices and culture. Researchers hoping to build on this piece might consider further examining the incentive structures that created the problems highlighted by Paullada and colleagues and, critically, working toward evidence-backed suggestions for how we might change these structures. Normative arguments can likely only take us so far. One approach might be in empowering the data-generating public,^9^ which may build on some of the legal avenues discussed by Paullada et al. Another opportunity to expand on the work in the paper is to continue engaging with more disciplinary perspectives; many of the issues discussed in the paper have been highlighted in other fields like economics and human-computer interaction.^10^ For machine learning to truly address some of the cultural issues outlined in the paper, it is critical that we break down the walls that have been constructed between our field and related areas.

## References

[1] Bandy J., Nichols J. (2021).

[2] Sambasivan N., Kapania S., Highfill H., Akrong D., Paritosh P., Aroyo L.M. (2021). CHI ’21: Proceedings of the 2021 CHI Conference on Human Factors in Computing Systems.

[3] Hecht B., Wilcox L., Bigham J.P., Schöning J., Hoque E., Ernst J. (2018). It’s Time to Do Something: Mitigating the Negative Impacts of Computing Through a Change to the Peer Review Process. ACM Future of Computing Blog.

[4] NeurIPS NeurIPs 2021 Datasets and Benchmarks Track. https://neurips.cc/Conferences/2021/CallForDatasetsBenchmarks.

[5] Gebru T., Morgenstern J., Vecchione B., Vaughan J.W., Wallach H., Daumé H., Crawford K. (2018). Datasheets for Datasets. arXiv.

[6] Olteanu A., Castillo C., Diaz F., Kıcıman E. (2019). Social data: Biases, methodological pitfalls, and ethical boundaries. Front Big Data.

[7] Prabhu V.U., Birhane A. (2020). Large image datasets: A pyrrhic win for computer vision?. arXiv.

[8] Paullada A., Raji I.D., Bender E.M., Denton E., Hanna A. (2021). Data and its (dis)contents: A survey of dataset development and use in machine learning research. Patterns.

[9] Vincent N., Li H., Tilly N., Chancellor S., Hecht B. (2021). FAccT ’21: Proceedings of the 2021 ACM Conference on Fairness, Accountability, and Transparency.

[10] Sen S., Lesicko M., Giesel M., Gold R., Hillman B., Naden S., Russell J., Wang Z., Hecht B. (2015). CSCW ’15: Proceedings of the 18th ACM Conference on Computer Supported Cooperative Work & Social Computing.

